# METTL3 Is Suppressed by Circular RNA circMETTL3/miR-34c-3p Signaling and Limits the Tumor Growth and Metastasis in Triple Negative Breast Cancer

**DOI:** 10.3389/fonc.2021.778132

**Published:** 2021-12-22

**Authors:** Han-guang Ruan, Wen-chao Gu, Wen Xia, Yan Gong, Xue-liang Zhou, Wen-yan Chen, Juan Xiong

**Affiliations:** ^1^ Department of Breast Oncology, The Third Hospital of Nanchang, Nanchang, China; ^2^ Department of Diagnostic of Radiology and Nuclear Medicine, Gunma University Graduate School of Medicine, Maebashi, Japan; ^3^ Department of Medical Oncology, State Key Laboratory of Oncology in South China, Collaborative Innovation Center for Cancer Medicine, Sun Yat-Sen University Cancer Center, Guangzhou, China; ^4^ Department of Cardiac Surgery, The First Affiliated Hospital, Nanchang University, Nanchang, China; ^5^ Department of Radiation Oncology, Jiangxi Cancer Hospital, Nanchang, China

**Keywords:** m^6^A, METTL3, miR-34c-3p, circMETTL3, triple negative breast cancer

## Abstract

Despite N6-methyladenosine (m^6^A) is functionally important in various biological processes, its role in the underlying regulatory mechanism in TNBC are lacking. In this study, we investigate the pathological role and the underlying mechanism of the m^6^A methylated RNA level and its major methyltransferase METTL3 in the TNBC progression. We found that the m^6^A methylated RNA was dramatically decreased in TNBC tissues and cell lines. Functionally, we demonstrated that METTL3 inhibits the proliferation, migration, and invasion ability of TNBC cells. Moreover, we found METTL3 is repressed by miR-34c-3p in TNBC cells. On the mechanism, we found that circMETTL3 could act as a sponge for miR-34c-3p and inhibits cell proliferation, invasion, tumor growth and metastasis by up-regulating the expression of miR-34c-3p target gene METTL3. In conclusion, our study demonstrates the functional importance and regulatory mechanism of METTL3 in suppressing the tumor growth of TNBC.

## Introduction

Breast cancer is the most common cancer among women. According to Global Cancer Statistics, there were 2,088,849 new cases and 626,679 deaths from breast cancer worldwide in 2018, accounting for nearly a quarter of all cancer cases among women worldwide (1, 2). Despite early detection and effective systematic treatment, breast cancer remains the leading cause of cancer death in more than 100 countries. Breast cancer is a heterogeneous disease, including four main subtypes (3). Triple negative breast cancer (TNBC) is a type of breast cancer with negative expression of estrogen receptor, progesterone receptor and human epidermal growth factor receptor 2 (4, 5). TNBC has special biological and clinicopathological features, such as strong proliferation and invasion ability, high recurrence and metastasis rate, and poor prognosis (6). The median survival time of patients with metastatic TNBC was only 13.3 months. Due to the absence of expression of ER, PR and HER2 in TNBC patients, effective endocrine therapy, and targeted therapy against HER2 are lacking (7). So far, TNBC is still dominated by chemotherapy (8, 9). Therefore, it is of great significance to study the molecular mechanisms of the occurrence and development of TNBC and to find effective potential targets for improving the survival and prognosis of TNBC patients.

Various epigenetic studies have focused on histone modifications, DNA methylation, and chromatin remodeling. Similarly, coding RNA nucleotides have a series of covalent modifications that manage gene expression by affecting RNA stability and translation (10). N6-methyladenosine (m^6^A), a kind of most abundant mRNA modifications, is transcriptome-wide presented in most RNAs and normally enriched near the 5′ UTRs (11–13). Emerging evidence shows that mammalian m6A is dynamically regulated and involved in various biological progress (14, 15). Especially, m^6^A was reported involved in several cancers. However, the pathological role and regulatory mechanism of this newly emerging RNA modification in TNBC have not been illustrated yet. Unveil its role in TNBC may help to develop new therapeutic strategies for TNBC patients.

The reversible m6A RNA modification is coordinated by a methyltransferase (m6A “writers”), m6A reader proteins, and demethylase (m6A “erasers”). These members cover more than 13 enzymes. The m^6^A “writers” complex containing METTL3, METTL14, WTAP, CBLL1, RBM15, ZC3H13, and VIRMA is responsible for the methylation of target RNA transcripts, including mRNA and non-coding RNAs (15–17). After which, m^6^A readers, including YTHDF1-3, YTHDC1, IGF2BPs, and eIF3, discern these m^6^A modifications to direct RNA alternative splicing, translation, localization, and RNA stability, among other processes (18). On the other hand, as the m^6^A “erasers,” such as FTO and ALKBH5 remove m^6^A modifications from the target transcripts (19–21). METTL3 is the major RNA methyltransferase implicated in mRNA biogenesis, decay, and translation control through m^6^A methylation. Recently, it is reported that METTL3 promotes the breast cancer progression *via* targeting Bcl-2 (22). On the other hand, low expression of METTL3 was associated with the poor prognosis of TNBC (23). Therefore, the underlying epigenetic regulation of METTL3 in TNBC has yet to be further investigated.

MicroRNAs (miRNAs) are a class of small non-coding RNAs that are evolutionarily conserved and regulate gene expression at the post-transcriptional level through targeted mRNA, leading to mRNA degradation or translation inhibition. The upregulation of oncogenic miRNAs resulted in decreased expression of tumor suppressor genes. Conversely, the down-regulation of tumor suppressor miRNAs increases the expression of oncogenes. Recent evidence proposed a hypothesis that competing endogenous RNA (ceRNA) suggested that RNAs including lncRNAs, circRNAs, mRNAs and Pseudogenes regulate each other through competing shared miRNAs, providing a new mechanism for gene expression regulation. Studies showed that more than 80% of circRNAs were derived from exons and were identical to the corresponding linear mRNA sequences (24, 25). Therefore, circRNA may, as a new member of ceRNA family, regulate miRNA activity by competing for common miRNA binding sites, and play an important role in the regulation of gene expression in tumors and other diseases (24, 25). More and more evidence indicate that circRNAs may be involved in the progression of breast, stomach, colorectal, bladder and hepatocellular carcinoma as ceRNAs (26–29). However, it is unclear which circRNAs may regulated the level of METTL3 in the tumorigenesis and tumor progression of TNBC.

In this study, we investigate the pathological role and the underlying mechanism of the m^6^A methylated RNA level and its major methyltransferase METTL3 in the TNBC progression. We found that the m^6^A methylated RNA was dramatically elevated in TNBC tissues and was associated with pathological grade, clinical stage, and poor prognosis. Functionally, we demonstrated that METTL3 promotes the proliferation, migration, and invasion ability of TNBC cells. Moreover, we found METTL3 is repressed by miR-34c-3p in TNBC cells. Further studies showed that circMETTL3 could act as a sponge for miR-34c-3p and promote cell proliferation, invasion, tumor growth and metastasis by up-regulating the expression of miR-34c-3p target gene METTL3. Therefore, circMETTL3 inhibits the tumor growth of triple negative breast cancer *via* miR-34c-3p/METTL3 signaling.

## Method and Materials

### Ethical Approval

Collection of 30 human TNBC and adjacent normal tissues was approved by the institutional review board of the third hospital of Nanchang city after obtaining written informed patient consent.

### Cell Culture

The human mammary epithelial (HME) cell lines (MCF10A and 184A1) and TNBC cell lines (MDA-MB-231 and BT-549) were purchased from the American Type Culture Collection company (Manassas, VA, USA) and respectively cultured in RPMI 1640 medium and DMEM medium (Thermo Fisher Scientific, Waltham, CA, USA) containing 10% fetal bovine serum (FBS; Thermo Fisher Scientific), 100 μg/ml penicillin and streptomycin. Cells were incubated at 37°C with 5% CO_2_.

### Global m^6^A Measurements

The level of global m6A in total RNA was quantified by EpiQuik m6A RNA Methylation Quantification Kit (Epigentek Group, Farmingdale, NY) as described in our previous study (30).

### m^6^A Dot Blot

m6A dot blot was performed to detect the qualitative m6A modifications as previously described (31). Briefly, poly-A RNA was purified by Dynabeads mRNA Purification Kit (Thermo Fisher, Carlsbad, CA, USA) and spotted on an Amersham Hybond-XL membrane. After an incubation with the anti-m6A primary antibody and the mouse-HRP secondary antibody, the membrane was incubated with Pierce ECL2 Western Blotting Substrate, and exposed to X-Ray Super RX Films.

### Cell Proliferation Assay

Cell proliferation was evaluated by Cell Counting Kit-8 assay (Dojindo Laboratories, Japan) (32). The cells (1×10^3^) were inoculated in a 96-well plate and incubated at 37°C for 24 h before transfection. CCK-8 solution (10 μL) was added to each well 48 h after transfection. After incubation at 37°C for 2 h, the absorbance was measured at 450 nm using Spectra Max 250 spectrophotometer (Molecular Devices, USA).

### Colony Formation Assay

The six-well plate was covered with 0.6% agar layer and 20% FBS was added to the medium. The cells (1×10^3^) were cultured with 0.3% agar for 2 weeks at 37°C. The number of colonies per well was fixed, stained with crystal violet, photographed, and counted.

### Wound Healing Assay

The evaluation of cell migration ability was performed by using wound scrape assay to examine the role of METTL3, miR-34c-3p and circMETTL3 in the regulation of migration ability of TNBC cell lines as described previously (33).

### Transwell Invasion Assay

The transwell culture system was performed to examine the invasive ability of METTL3, miR-34c-3p and circMETTL3 on TNBC cell lines as described previously (33).

### Quantitative Real-Time PCR (qRT-PCR)

A total of 1 μg extracted RNA was transcribed into cDNA and qRT-PCR was performed to evaluate the expression level by ABI ViiA 7 Real-Time PCR System (Applied Biosystems) as described in our previous study (30). GAPDH was used as an internal control. The specific primers sequence was showed as follows:

**Table:** 

Gene	Forward primer (5’-3’)	Reverse primer (5’-3’)
METTL3	CAAGCTGCACTTCAGACGAA-	GCTTGGCGTGTGGTCTTT
METTL14	CTACCCATCCTCACTGTCAGTC	GGATGTTCCTGTTTGACCTGAGG
RBM15	TCCCACCTTGTGAGTTCTCC	GTCAGCGCCAAGTTTTCTCT
WTAP	CTTCCCAAGAAGGTTCGATTGA	TCAGACTCTCTTAGGCCAGTTAC
VIRMA	AATCCTGTGGGAAGATCAGC	ACACGTAAGGCAGTGGTAAG
FTO	CCAGAACCTGAGGAGAGAATGG	CGATGTCTGTGAGGTCAAACGG
ALKBH	CCAGCTATGCTTCAGATCGCCT	GGTTCTCTTCCTTGTCCATCTCC
GAPDH	TGACTTCAACAGCGACACCCA	CACCCTGTTGCTGTAGCCAAA

### Western Blot

Western Blot was performed as described in our previous study (32). Briefly, protein was isolated from TNBC tissues or cells, separated by 12% SDS-PAGE and transferred to PVDF membranes. Membranes were then probed with the primary antibodies as follows: anti-METTL3 (Abcam, Cambridge, UK) and anti-GAPDH (Santa Cruz Biotechnology).

### Dual-Luciferase Report Assay

Luciferase reporter vectors and mutants with full length circMETTL3 or METTL 3 ‘-UTR were constructed. MDA-MB-231 cells were transfected with luciferase reporter vector miR-34c-3p mimic or miR-34c-3p inhibitor. After 48 hours of culture, luciferase activity in firefly and renal tubule was quantified by the double luciferase report test (Promega, USA).

### Xenograft Model

All animal studies were approved by the Animal Protection and Use Committee of the third hospital of Nanchang. Follow standard animal care and laboratory guidelines according to the protocol. 4 weeks old female BALB/C nude mice were subcutaneously injected with 5×10^6^ cancer cells (5 in each group). Tumor volume was measured every 5 days for 30 consecutive days, and the calculation formula was: volume = length × width^2^/2.

### Statistical Analysis

The data set used is from GEO database (https://www.ncbi.nlm.nih.gov/geo/), and the download data format is MINIML. Box plots are drawn by boxplot; PCA graphs are drawn by R software package ggord; The box plot is implemented by the R software package ggplot2; the heat map is displayed by the R software package pheatmap. Raw counts of RNA-sequencing data (level 3) from TNBC patients were obtained from The Cancer Genome Atlas (TCGA) dataset (https://portal.gdc.cancer.gov/), in which the method of acquisition and application complied with the guidelines and policies. The KM survival analysis with log-rank test were also used to compare the survival difference between above two groups or more groups. TimeROC analysis was performed to compare the predictive accuracy of each gene and risk score. For Kaplan–Meier curves, p-values and hazard ratio (HR) with 95% confidence interval (CI) were generated by log-rank tests and univariate Cox proportional hazards regression. All analytical methods above and R packages were performed using R software version v4.0.3 (The R Foundation for Statistical Computing, 2020). Data were expressed as the Means ± SEM. For two groups, 2-tailed t-test (unpaired) was used for comparisons. For multiple comparisons, ANOVA followed by the *post hoc* Bonferroni test was taken with GraphPad Prism^®^ version 6.0 software (GraphPad Software, Inc., La Jolla, CA, USA).

## Results

### Expression of N6-Methyladenosine Methyltransferase METTL3 and m6A Level Is Decreased in TNBC Tissues and Cell Lines

**Figure 1:**
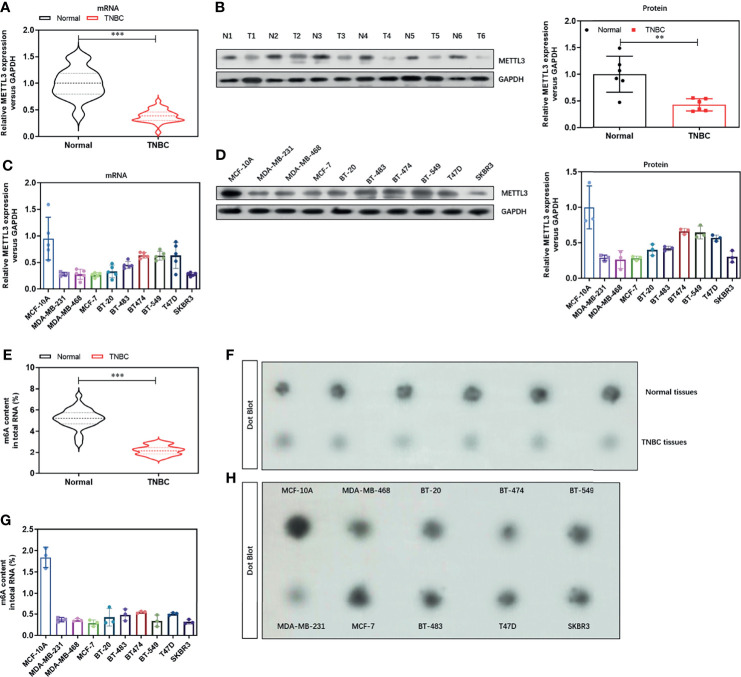
Expression of N6-methyladenosine methyltransferase METTL3 and m6A level is decreased in TNBC tissues and cell lines. **(A)** The mRNA level of METTL3 in TNBC tissues and paired normal tissues were detected by real-time PCR (n=30). **(B)** The protein level of METTL3 in TNBC tissues and paired normal tissues were detected by Western Blot (n=6). **(C)** The mRNA level of METTL3 in 10 breast cell lines, including 9 breast cancer cell lines (MDA-MB-231, MDA-MB-469, MCF-7, BT-20, BT-483, BT-474, BT-549, T47D and SKBR3) and one HME cell lines (MCF-10A) were detected by real-time PCR. **(D)** The protein level of METTL3 in 10 breast cell lines were confirmed by Western Blot. **(E)** The global m^6^A RNA level in TNBC tissues and paired normal tissues were detected by Colorimetric m^6^A RNA Methylation Assay Kit (n=30). **(F)** The global m^6^A RNA level in TNBC tissues and paired normal tissues were confirmed by Dot Blot (n=6). **(G)** The global m^6^A RNA level in 10 breast cell lines were detected by Colorimetric m^6^A RNA Methylation Assay Kit. **(H)** The global m^6^A RNA level in 10 breast cell lines were confirmed by Dot Blot. **P < 0.01 and ***P < 0.001 versus indicated group.

To analyzed the potential role of the major methyltransferase METTL3 in the TNBC progression. We evaluated the mRNA levels in 30 cases of TNBC tissues and found a significant decrease **(** **Figure 1A** **)**, compared with paired normal tissues. We then grouped these tissues according to the clinical characterizes including age, menopause, tumor size, histopathological grade, lymph node metastasis, distant metastasis, and histopathological grade. We found that METTL3 was negatively correlated with distant metastasis and histopathological grade (**Supplementary Table 1**). We further verified the decreased protein level of METTL3 in the 6 paired TNBC tissues and normal tissues **(** **Figure 1B** **)**. We next confirmed the expression level of METTL3 in 10 breast cell lines, including 9 breast cancer cell lines (MDA-MB-231, MDA-MB-469, MCF-7, BT-20, BT-483, BT-474, BT-549, T47D and SKBR3) and one HME cell lines (MCF-10A). As shown in **Figures 1C, D**, the mRNA **(** **Figure 1C** **)** and protein **(** **Figure 1D** **)** levels of METTL3 were dramatically decreased in the breast cancer cell lines, especially in the TNBC cell lines. We then evaluated the global m^6^A methylated RNA level in the TNBC tissues and cell lines. Consistently, the global m^6^A methylated RNA level was also lower in the TNBC tissues **(** **Figure 1E** **)** and breast cancer cell lines **(** **Figure 1G** **)**, than the normal tissues and MCF-10A cell lines, respectively. These increasing was further confirmed by Dot Blot assay, as shown in **Figures 1F, H**. We further analyzed the expression pattern of METTL3 in normal tissues and TNBC tissues from GEO database (GSE38959) and found a lower level of METTL3 in the TNBC tissues (**Supplementary Figure 1** **, P<0.01**). Moreover, the prognostic analysis of METTL3 expression in TNBC tissues in the TCGA set showed that patients with low level of METTL3 seems to have a poorer prognostic (**Supplementary Figure 2**). These data demonstrate that METTL3 may be critical to the progression of TNBC.

### Over-Expression of METTL3 Inhibits Proliferation, Migration, and Invasion in TNBC Cells

**Figure 2:**
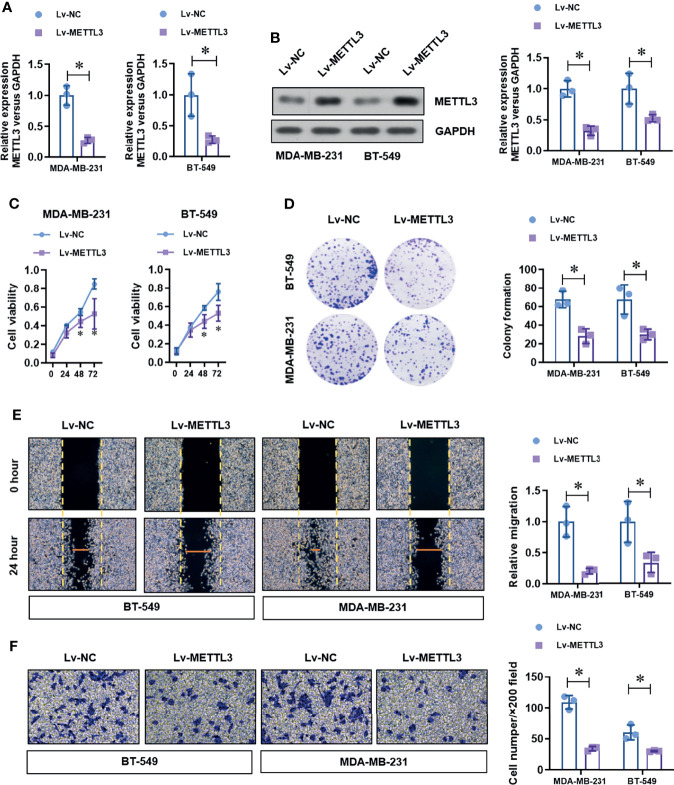
Over-expression of METTL3 inhibits proliferation, migration, and invasion in TNBC cells. **(A)** The over-expression efficiency of METTL3 was analyzed by real-time PCR. **(B)** The over-expression efficiency of METTL3 was confirmed by Western Blot. **(C)** The cell viability of normal or METTL3 over-expressed TNBC cells was detected by CCK-8 assay. **(D)** The cell proliferation of normal or METTL3 over-expressed TNBC cells was detected by colony formation assay. **(E)** The migration ability of normal or METTL3 over-expressed TNBC cells was detected by wound healing assay. **(F)** The invasion ability of normal or METTL3 over-expressed TNBC cells was detected by transwell assay. *P < 0.05 versus indicated group.

To determine the critical role of METTL3 in TNBC cells, we over-expressed the level of METTL3 by transfecting the specific shRNA vectors target METTL3. The efficiency of METTL3 over-expression was verified by real-time PCR and Western blot. As shown in **Figures 2A, B**, the mRNA and protein level of METTL3 was obviously increased by METTL3 over-expression We then detected the effect of METTL3 over-expression on the cell proliferation ability. METTL3 over-expression significantly decreased the cell viability **(** **Figure 2C** **)** and colony formation ability **(** **Figure 2D** **)** by CCK-8 assay and colony formation assay. Moreover, the wound healing assay and Transwell assay demonstrated that METTL3 over-expression significantly impaired the migration **(** **Figure 2E** **)** and invasion **(** **Figure 2F** **)** abilities of TNBC cell lines. These results suggest a tumor suppressor role of METTL3 in the TNBC cells.

### METTL3 Is Directly Targeted by miR-34c-3p

**Figure 3:**
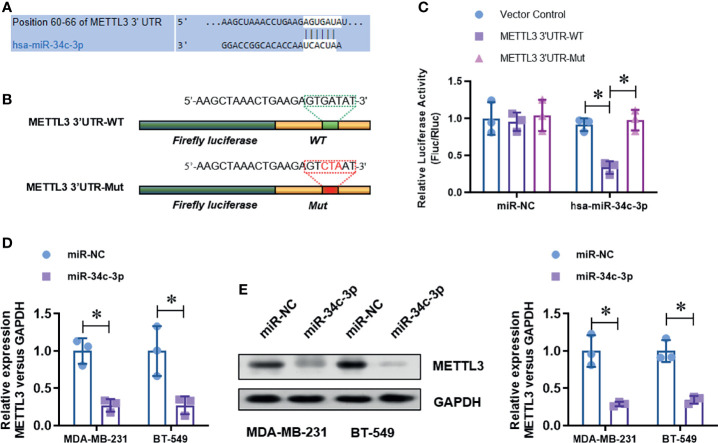
METTL3 is directly targeted by miR-34c-3p. **(A)** The putative bind site between METTL3 3’UTR and miR-34c-3p was predicted by Target Scan tool. **(B)** Two dual-luciferase reporter vectors containing wild-type METTL3 3’-UTR (METTL3 3’-UTR-WT) or site mutant METTL3 3’-UTR (METTL3 3’-UTR-Mut) were constructed. **(C)** Dual-luciferase assay was used to evaluate the targeting of miR-34c-3p to wild-type METTL3 3’-UTR. **(D)** The level of METTL3 mRNA in normal or miR-34c-3p over-expressed TNBC cells was detected by real-time PCR. **(E)** The level of METTL3 protein in normal or miR-34c-3p over-expressed TNBC cells was detected by Western Blot. *P < 0.05 versus indicated group.

**Figure 4:**
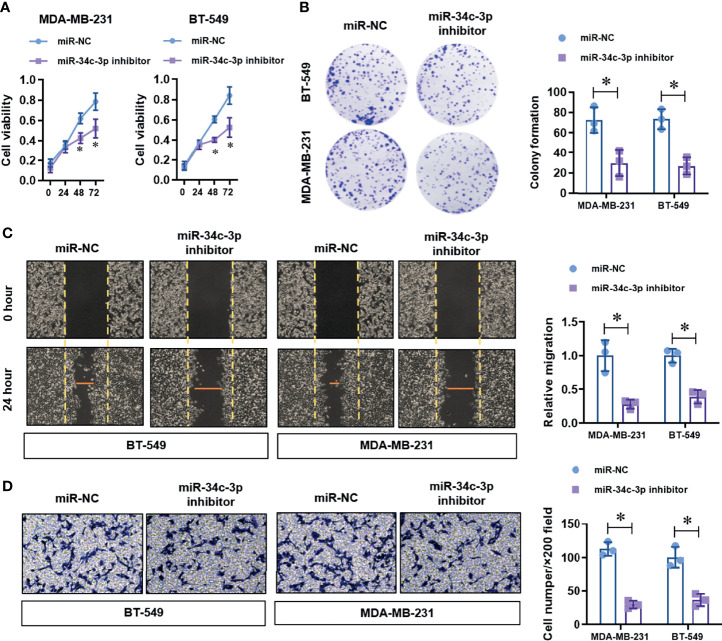
MiR-34c-3p inhibition impairs proliferation, migration, and invasion in TNBC cells. **(A)** The cell viability of normal or miR-34c-3p inhibited TNBC cells was detected by CCK-8 assay. **(B)** The cell proliferation of normal or miR-34c-3p inhibited TNBC cells was detected by colony formation assay. **(C)** The migration ability of normal or miR-34c-3p inhibited TNBC cells was detected by wound healing assay. **(D)** The invasion ability of normal or miR-34c-3p inhibited TNBC cells was detected by transwell assay. *P < 0.05 versus indicated group.

To identify the up-stream regulator of METTL3 in TNBC cells, we screened the potential miRNAs in the on-line database (Target Scan and Star Base). We found that miR-34c-3p is highly conserved targeting the 3’-UTR sequences of METTL3 mRNA (position of 60-66bp), as shown in the **Figure 3A**. To verified the targeting of miR-34c-3p to METTL3 3’-UTR. We constructed two dual-luciferase reporter vectors containing wild-type METTL3 3’-UTR (METTL3 3’-UTR-WT) or site mutant METTL3 3’-UTR (METTL3 3’-UTR-Mut) (**Figure 3B**). After co-transfection with miR-NC or miR-34c-3p mimics in MDA-MB-231 cells, we detected the luciferase activity and found a dramatically decrease in the METTL3 3’-UTR-WT group with miR-34c-3p transfection, which is unchanged in the METTL3 3’-UTR-Mut group (**Figure 3C**). Moreover, miR-34c-3p over-expression significantly suppressed the mRNA (**Figure 3D**) and protein (**Figure 3E**) level of METTL3 in MDA-MB-231 and BT-549 cells. To determine the critical role of miR-34c-3p in TNBC cells, we then evaluated the effect of miR-34c-3p on cell proliferation, migration and invasion abilities of TNBCs. As shown in **Figure 4A**, miR-34c-3p inhibition largely impaired the cell proliferation (**Figures 4A, B**), migration (**Figure 4C**) and invasion (**Figure 4D**) abilities of TNBCs. These results suggest that METTL3 is directly targeted by miR-34c-3p in the TNBC cells.

### CircMETTL3 Serves as a Sponge for miR-34c-3p

**Figure 5:**
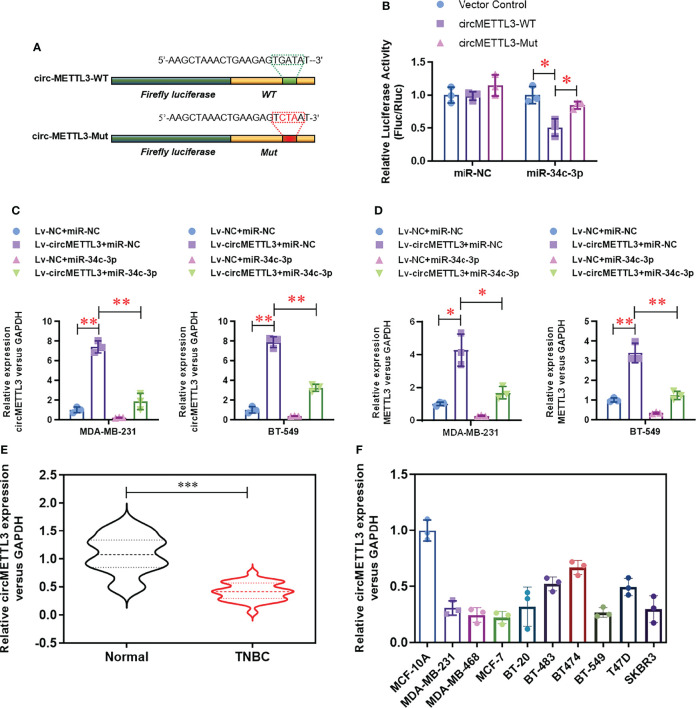
CircMETTL3 serves as a sponge for miR-34c-3p. **(A)** Two dual-luciferase reporter vectors containing wild-type circMETTL3 (circMETTL3-WT) or site mutant circMETTL3 (circMETTL3-Mut) were constructed. **(B)** Dual-luciferase assay was used to evaluate the targeting of miR-34c-3p to wild-type circMETTL3. **(C)** The over-expression of circMETTL3 was evaluated by real-time PCR in the indicated TNBC cells. **(D)** The level of METTL3 mRNA was evaluated by real-time PCR in the indicated TNBC cells. **(E)** The level of circMETTL3 in TNBC tissues and paired normal tissues were detected by real-time PCR (n=30). **(F)** The level of circMETTL3 in 10 breast cell lines were detected by real-time PCR. *P < 0.05, **P < 0.01 and ***P < 0.001 versus indicated group.

To further identify the potential up-stream sponge circular RNAs for miR-34c-3p in TNBCs, we found a highly enriched circMETTL3 (hsa_circ_0101463) with miR-34c-3p. Notably, circMETTL3 is derived from alterative splicing of the same genome and share the similar miR-34c-3p binding sequence with METTL3. Dual-luciferase assay (**Figure 5A**) demonstrated that the luciferase activity of wild-type circMETTL3 reporter vector was obviously decreased by miR-34c-3p, which is not appears in the mutant circMETTL3 reporter vector group (**Figure 5B**). Over-expression of circMETTL3 (**Figure 5C**) dramatically up-regulated the expression level of METTL3 levels (**Figure 5D**), which was reversed by miR-34c-3p. Moreover, the level of circMETTL3 was highly expressed in the TNBC tissues (**Figure 5E**) and cell lines (**Figure 5F**), similar with the expression pattern of METTL3.

### Over-Expression of CircMETTL3 Inhibits Proliferation, Migration, and Invasion *via* miR-34c-3p/METTL3 Signaling Pathway

**Figure 6:**
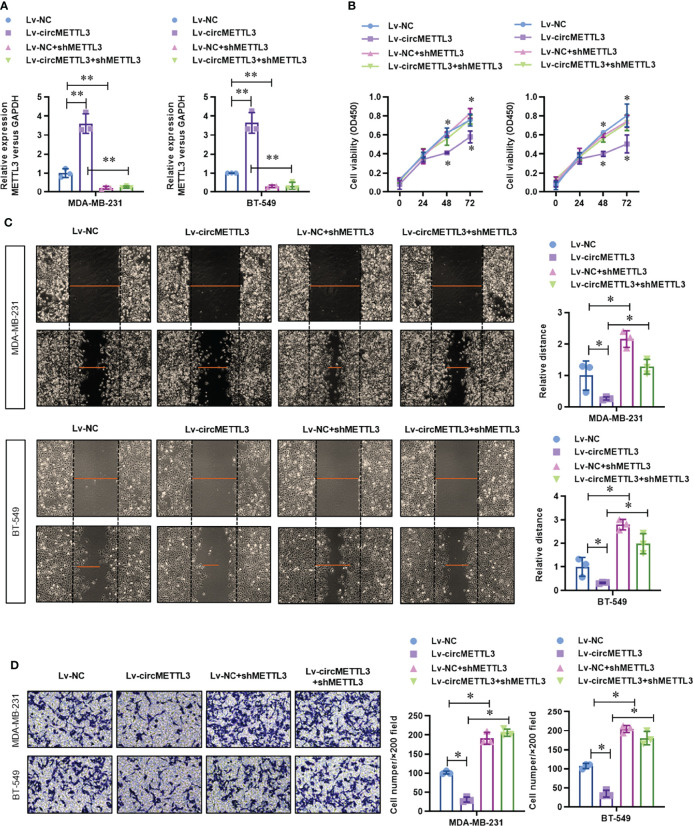
Over-expression of CircMETTL3 inhibits proliferation, migration, and invasion *via* miR-34c-3p/METTL3 signaling pathway *in vitro*. **(A)** The level of METTL3 mRNA in the circMETTL3 over-expression with or without METTL3 knock-down TNBC cells was evaluated by real-time PCR. **(B)** The cell viability of circMETTL3 over-expression with or without METTL3 knock-down TNBC cells was detected by CCK-8 assay. **(C)** The migration ability of circMETTL3 over-expression with or without METTL3 knock-down TNBC cells was detected by wound healing assay. **(D)** The invasion ability of circMETTL3 over-expression with or without METTL3 knock-down TNBC cells was detected by transwell assay. *P < 0.05 versus indicated group.

**Figure 7:**
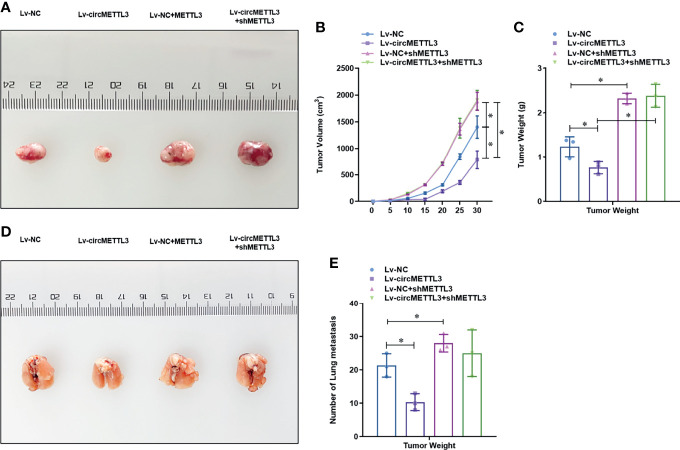
Over-expression of CircMETTL3 inhibits the tumor growth and metastasis of MDA-MB-231 cells *via* miR-34c-3p/METTL3 signaling pathway *in vivo*. **(A)** *In vivo* xenograft models were performed and representative image of generated tumors were shown. **(B)** Tumor volume was monitored every 5 days for 30 days; **(C)** The tumor weight was analyzed after the mice was sacrificed; **(D)** *In vivo* xenograft models were performed and representative image of lung metastasis were shown; **(E)** The number of lung metastasis were counted. *P < 0.05 versus indicated group.

To determine the functional role of circMETTL3, we knock-downed the level of METTL3 in the normal or circMETTL3 over-expressed MDA-MB-231 and BT-549 cells. CircMETTL3 over-expression obviously increased the level of METTL3 mRNA in TNBC cells, which is reversed by METTL3 knock-down (**Figure 6A**). Moreover, circMETTL3 over-expression significantly inhibited the proliferation ability of TNBC cells, indicated by CCK-8 assay (**Figure 6B**). Similarly, circMETTL3 over-expression significantly inhibited the migration and invasion ability of TNBC cells, indicated by wound healing assay (**Figure 6C**) and transwell assay (**Figure 6D**). However, these inhibitions were totally reversed by METTL3 knock-down (**Figures 6B–D**). Furthermore, we generated a xenograft model of TNBC in BALB/c NOD mice to test the functional role of circMETTL3 and METTL3 *in vivo*. CircMETTL3 over-expression obviously decreased tumor volume growth (**Figures 7A–C**) and lung metastasis (**Figures 7D, E** **)** of MDA-MB-231 cells. Moreover, These decreases were also totally reversed by METTL3 knock-down. These results collectively demonstrates that over-expression of CircMETTL3 inhibits proliferation, migration, and invasion *via* miR-34c-3p/METTL3 signaling pathway.

## Discussions

Post-transcriptional modification has become an important regulator of many physiological processes and disease progression and has attracted more and more attention in biological science research. Among the many RNA modifications, m^6^A is the most abundant mRNA modification. An average of 1,000 nucleotides contains one or two m^6^A residues. It can be installed and removed by methyltransferase complex and alters the expression of target genes, thereby affecting the corresponding cellular processes and physiological functions. In terms of molecular mechanisms, m^6^A is involved in almost all steps of RNA metabolism, including mRNA translation, degradation, splicing, output, and folding. The role of m^6^A in various cancers has recently been reported. Jie Wu et al. proposed a ten-m6A-related LncRNAs as potential biomarkers to predict the prognostic risk of TNBC (34). Jaclyn M Einstein et al. found that inhibition of YTHDF2 triggers proteotoxic cell death in MYC-driven breast cancer (35). However, rarely studies reported whether m^6^A methylation contributes to cell proliferation and metastasis in TNBC, and what underlying mechanisms are involved. As the major RNA methyltransferase, METTL3 catalyze m^6^A methylation in numerous RNAs (36, 37). Recently, Wang et al. found that the m6A regulator genes including ALKBH5, YTHDF2, HNRNPC, KIAA1429, and RBM15 were significantly up-regulated in TNBC tissues, whereas some other regulators including FTO, YTHDC1, YTHDC2, METTL3, METTL14, and ZC3H13 were significantly down-regulated (38). Jun Shi reported that reduced expression of METTL3 promotes metastasis of TNBC by m6A methylation-mediated COL3A1 up-regulation (23). However, the roles of METTL3 and its upstream singling in the progression of TNBC are not well understood. Herein, we study this question in TNBC progression and found that METTL3 is dramatically decreased in TNBC tissues and cell lines, which over-expression significantly impairs the proliferation, migration, and invasion ability. Our findings initially unveiled the role of METTL3 in TNBC, and theoretically, it suggests that METTL3 may be a critical tumor suppressor of TNBC.

Many miRNAs and lncRNAs have been reported to regulate TNBC. However, it is not clear whether circRNAs play a role in the regulation of METTL3 in TNBC. Based on abundant evidence, the ceRNA hypothesis describes how RNA communicates with miRNAs by competing to bind to and regulate each other’s expression, thus constructing complex post-transcriptional regulatory networks. miR-34c-3p has been reported to regulate tumor progression in many cancers. In prostate cancer, miR-34c-3p negatively regulates CD44 and inhibits tumor regeneration and metastasis. In glioblastoma, miR-34c-3p has been identified as a tumor suppressor due to its regulation of the TGF-β signaling network. In colon cancer, miR-34c-3p inhibits self-renewal and differentiation of tumor stem cells by targeting Notch1. On the mechanism, we illustrated that METTL3 is a downstream target of miR-34c-3p in TNBC progression. Because of the important role of miR-34c-3p in cancer, mir-34-targeted gene therapy has been encouraged for many types of cancer. In addition, we found that circMETTL3 was down-regulated in TNBC and act as a sponge of miR-34c-3p in TNBC cells. Further experiments showed that circMETTL3 could impaired TNBC proliferation, migration, and invasion *via* miR-34c-3p/METTL3 signaling.

These results collectively suggest that circMETTL3 plays an important tumor suppressor role in TNBC progression *via* miR-34c-3p/METTL3 signaling and may be a potential prognostic biomarker and therapeutic target for TNBC.

## Data Availability Statement

The raw data supporting the conclusions of this article will be made available by the authors, without undue reservation.

## Ethics Statement

The studies involving human participants were reviewed and approved by the institutional review board of the third hospital of Nanchang city. The patients/participants provided their written informed consent to participate in this study. The animal study was reviewed and approved by the Animal Protection and Use Committee of the third hospital of Nanchang.

## Author Contributions

JX and H-gR designed this study. H-gR, W-cG, and WX performed the experiments. YG, X-lZ, and W-yC analyzed the data. JX and H-gR drafted the manuscript. All authors contributed to the article and approved the submitted version.

## Conflict of Interest

The authors declare that the research was conducted in the absence of any commercial or financial relationships that could be construed as a potential conflict of interest.

## Publisher’s Note

All claims expressed in this article are solely those of the authors and do not necessarily represent those of their affiliated organizations, or those of the publisher, the editors and the reviewers. Any product that may be evaluated in this article, or claim that may be made by its manufacturer, is not guaranteed or endorsed by the publisher.
