# Supplementary material for: METTL3 Is Suppressed by Circular RNA circMETTL3/miR-34c-3p Signaling and Limits the Tumor Growth and Metastasis in Triple Negative Breast Cancer
Source: Front Oncol. 2021 Dec 22;11:778132. doi: 10.3389/fonc.2021.778132 (PMC8727604; doi:10.3389/fonc.2021.778132)
Supplement: Supplementary file 1 [file DataSheet_1.docx]

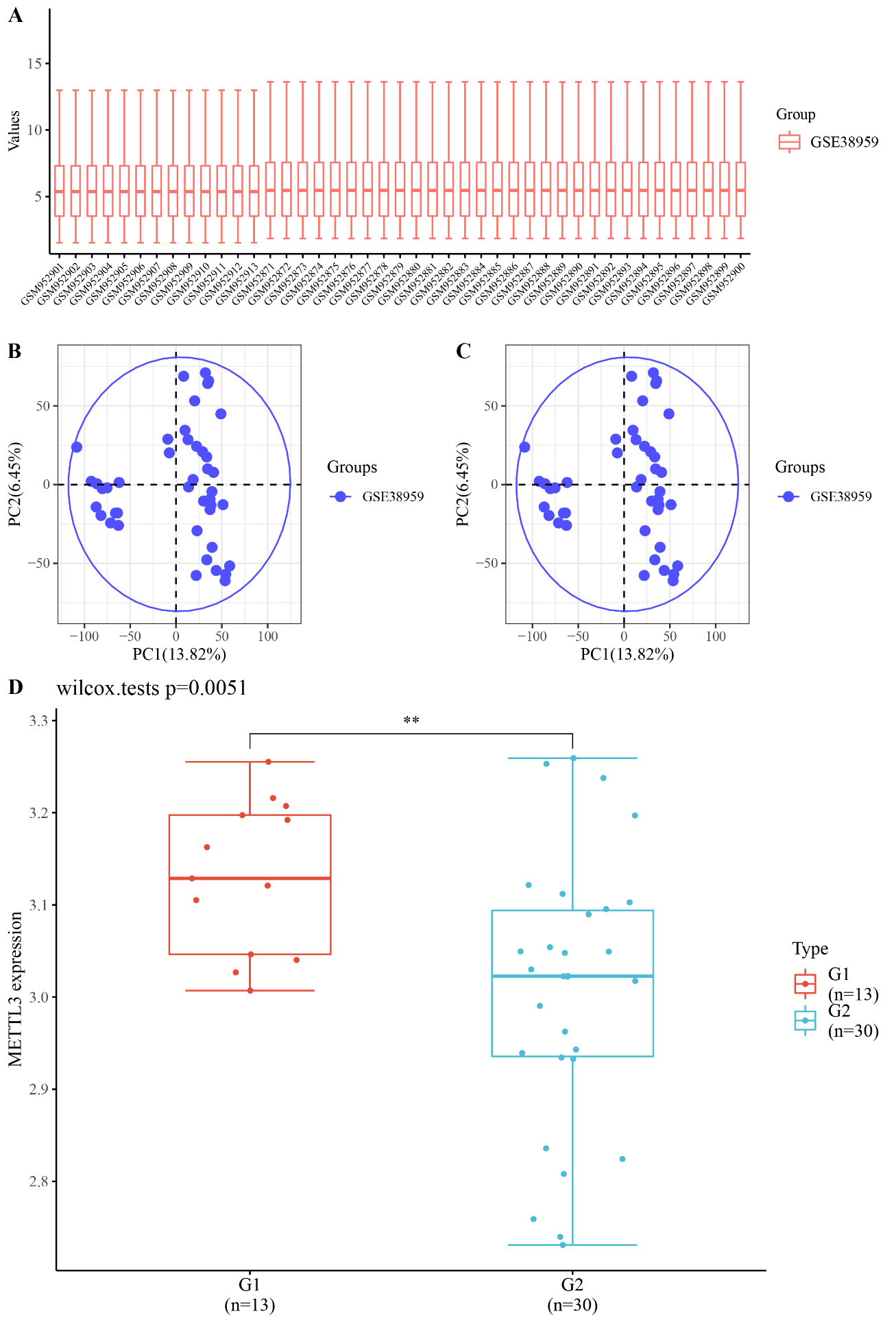


**Supplementary Figure 1. METTL3 expression difference between normal tissues and TNBC tissues from GEO database (GSE38959).** (A) Box plot after data standardization, different colors represent different data sets. (B) PCA results before batch removal for multiple data sets; (C) PCA results after batch removal. (D) The expression distribution of METTL3 in normal tissues (n=13) and TNBC tissues (n=30). ***P*<0.01.


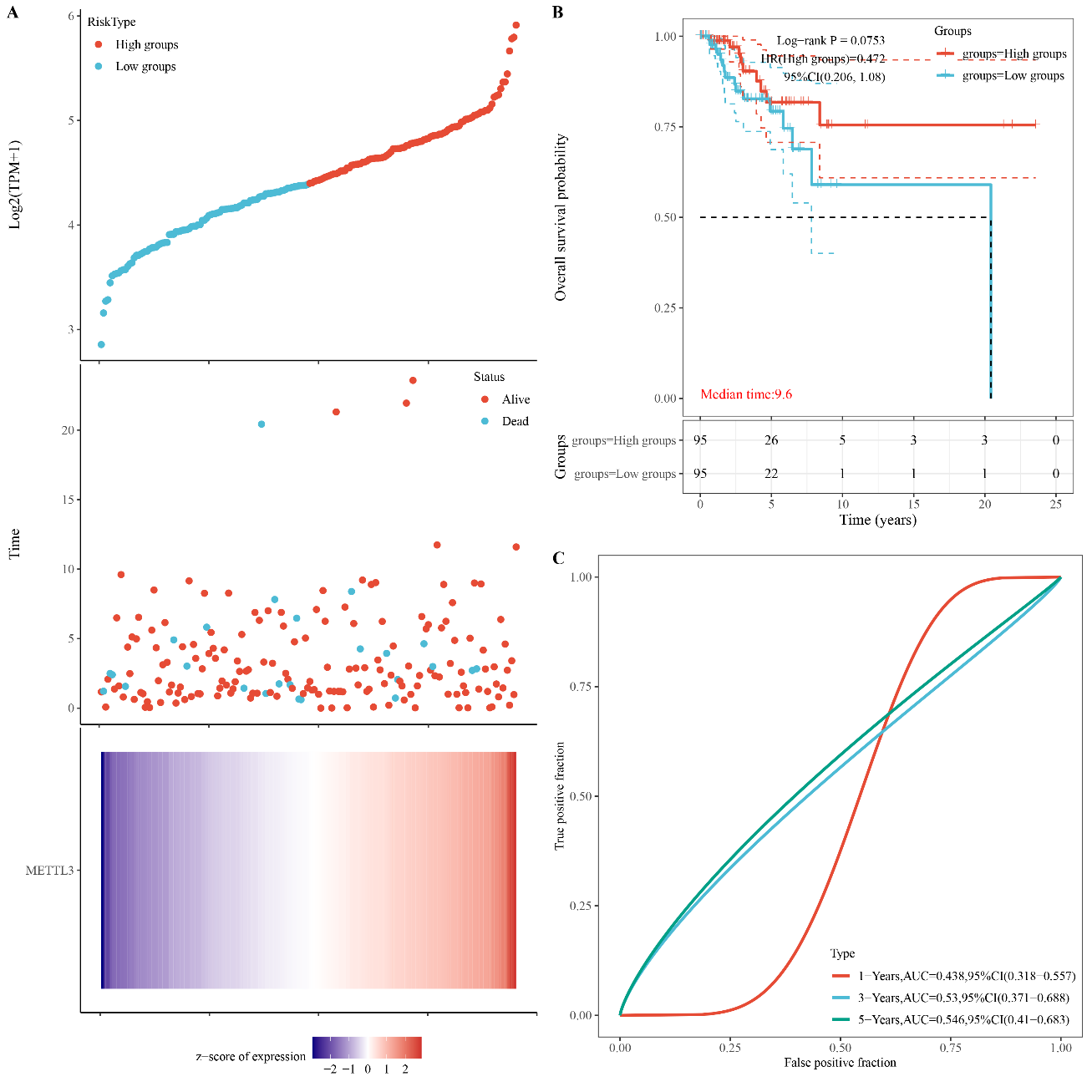


**Supplementary Figure 2. Prognostic analysis of METTL3 expression in TNBC tissues in the TCGA set.** The dotted line represented the median risk score and divided the patients into low-risk and high-risk group. (A) The curve of risk score. Survival status of the patients. More dead patients corresponding to the higher risk score. Heatmap of the expression profiles of the five prognostic genes in low- and high-risk group. (B) Kaplan-Meier survival analysis of METTK3 signature. (C) Time-dependent ROC analysis the of METTL3. ROC receiver operating characteristic.
