# Supplementary material for: METTL3 Is Suppressed by Circular RNA circMETTL3/miR-34c-3p Signaling and Limits the Tumor Growth and Metastasis in Triple Negative Breast Cancer
Source: Front Oncol. 2021 Dec 22;11:778132. doi: 10.3389/fonc.2021.778132 (PMC8727604; doi:10.3389/fonc.2021.778132)
Supplement: Supplementary file 2 [file Table_1.docx]

**Supplementary table 1. Relationship between METTL3 expression and pathological features in TNBC patients.**

| Variables | | Tumor METTL3 Expression | | P Value |
| --- | --- | --- | --- | --- |
|  |  | High | Low |  |
| Age (years) | ≤50 | 9 | 10 | 0.358 |
|  | ＞50 | 5 | 6 |  |
| Menopause | Yes | 3 | 3 | 0.869 |
|  | No | 11 | 13 |  |
| Tumor Size | ≤2cm | 8 | 9 | 0.436 |
|  | >2cm | 7 | 6 |  |
| Lymph node metastasis | Yes | 2 | 4 | 0.233 |
|  | No | 11 | 13 |  |
| Distant metastasis | Yes | 4 | 6 | 0.021* |
|  | No | 7 | 13 |  |
| Grade | G1 | 3 | 4 | 0.034* |
|  | G2-3 | 10 | 13 |  |

**P*<0.05
